# Transforming Tomato Industry By-Products into Antifungal Peptides Through Enzymatic Hydrolysis

**DOI:** 10.3390/ijms26157438

**Published:** 2025-08-01

**Authors:** Davide Emide, Lorenzo Periccioli, Matias Pasquali, Barbara Scaglia, Stefano De Benedetti, Alessio Scarafoni, Chiara Magni

**Affiliations:** 1 Department of Food, Environmental and Nutritional Sciences (DeFENS), Università degli Studi di Milano, Via G. Celoria 2, 20133 Milan, Italy; davide.emide@unimi.it (D.E.); lorenzo.periccioli@unimi.it (L.P.); matias.pasquali@unimi.it (M.P.); stefano.debenedetti@unimi.it (S.D.B.); alessio.scarafoni@unimi.it (A.S.); 2 Department of Industrial Engineering (DII), University of Padova, Via Gradenigo 6/A, 35100 Padova, Italy; 3 Department of Agricultural and Environmental Sciences Production, Landscape, Agroenergy (DISAA), University of Milan, Via Celoria 2, 20133 Milan, Italy; barbara.scaglia@unimi.it

**Keywords:** tomato seed, agri-food by-products, enzymatic hydrolysis, antioxidant peptides, antifungal activity, circular economy, sustainable agriculture

## Abstract

In the context of the valorization of agri-food by-products, tomato (*Solanum lycopersicum* L.) seeds represent a protein-rich matrix containing potential bioactives. The aim of the present work is to develop a biochemical pipeline for (i) achieving high protein recovery from tomato seed, (ii) optimizing the hydrolysis with different proteases, and (iii) characterizing the resulting peptides. This approach was instrumental for obtaining and selecting the most promising peptide mixture to test for antifungal activity. To this purpose, proteins from an alkaline extraction were treated with bromelain, papain, and pancreatin, and the resulting hydrolysates were assessed for their protein/peptide profiles via SDS-PAGE, SEC-HPLC, and RP-HPLC. Bromelain hydrolysate was selected for antifungal tests due to its greater quantity of peptides, in a broader spectrum of molecular weights and polarity/hydrophobicity profiles, and higher DPPH radical scavenging activity, although all hydrolysates exhibited antioxidant properties. In vitro assays demonstrated that the bromelain-digested proteins inhibited the growth of *Fusarium graminearum* and *F. oxysporum* f.sp. *lycopersici* in a dose-dependent manner, with a greater effect at a concentration of 0.1 mg/mL. The findings highlight that the enzymatic hydrolysis of tomato seed protein represents a promising strategy for converting food by-products into bioactive agents with agronomic applications, supporting sustainable biotechnology and circular economy strategies.

## 1. Introduction

The recovery of bioactive compounds from food processing waste represents both an opportunity and a challenge for the agri-food sector. Industries operating in the fields of nutraceuticals, food packaging, and sustainable agriculture stand to benefit significantly from such innovations. Moreover, EU policies play a pivotal role in regulating food waste and by-product management, promoting a sustainable circular economy through waste reduction and improved resource efficiency [1,2,3,4]. The agri-food industry, which plays a crucial role in ensuring global nutrition, generates significant quantities of by-products, often regarded as waste. This leads to both economic and environmental challenges. Recovering bioactive compounds from plant-based by-products has emerged as a key strategy for developing sustainable biotechnological processes within the framework of the circular economy [5,6]. By-products are secondary materials generated during processing that still hold potential value. In contrast, waste refers to materials discarded because they are no longer useful, often due to spoilage, contamination, or overproduction. Both arise along the food supply chain, from primary production to distribution [1]. Agri-food by-products are still rich in compounds with potential applications in biostimulation, green pesticides, and nutrition. However, realizing this potential often requires appropriate chemical and physical treatments. The revalorization of agri-food waste biomass not only enables the production of high-value products but also addresses the critical issue of waste accumulation [7], highlighting the need for thorough economic evaluation in the development of such processes.

A key barrier to the recovery of bioactive compounds is the matrix effect, arising from the structural complexity of plant tissues and further intensified by industrial processing. This effect can reduce extraction efficiency and selectivity while promoting the co-extraction of unwanted compounds, such as lipids and polysaccharides. Moreover, interactions between bioactives and their native matrices can limit bioavailability and biological activity. Overcoming these limitations requires tailored extraction strategies, including enzymatic or physicochemical treatments, to improve yields while preserving bioactivity [8].

Tomato (*Solanum lycopersicum* L.) is a widely cultivated fruit, with global production reaching 182.3 million tons in 2018. China leads the world in tomato production, accounting for approximately 31.8% of the worldwide total. Other major producers include India, Turkey, the United States, and Mexico [9,10]. In Italy, tomato is one of the most developed horticultural commodities. Its processing produces a huge amount of tomato pomace, with seeds comprising 60% and peels 40% [10], which remains underexploited and scarcely valorized.

Tomato peel is considered a source of lycopene, as well as several other potentially bioactive compounds, including flavonoids [11,12]. Tomato seeds represent the primary source of protein, offering a valuable source of potential bioactive peptides [13]. Upon enzymatic digestion, seed storage proteins can release different classes of peptides with antioxidant, anti-inflammatory, or antimicrobial properties—potentially useful for both human health and crop protection applications.

Tomato is among the crops susceptible to *Fusarium* infections. Management of *Fusarium* pathogens requires the integration of approaches and benefits from the use of innovative strategies to control the diseases they cause. *F. oxysporum* f.sp. *lycopersici* is one of the major pathogens on tomato, causing wilt rot on the plant [14] while *F. graminearum* is a major pathogen of wheat and maize, causing direct damage and contamination of grains with mycotoxins, making it one of the most important fungal threats to agriculture. *Fusarium* species can be controlled with chemical approaches, but the limitation is that few classes of effective molecules are still on the market, and the rise in resistance phenomena [14,15]. The use of novel, naturally derived molecules to control them is crucial for the future of crop protection. Other agri-food by-products, such as okara from soy (*Glycine max*), have already demonstrated their potential in the context of crop protection against pathogenic fungi [16]. The potential use of plant-derived peptides represents a valuable strategy to control plant pathogens [17].

In this context, the present work aimed to develop a biochemical pipeline for the valorization of tomato by-products through alkaline extraction, selective enzymatic hydrolysis, and characterization of the resulting peptides. Three food-grade proteases, namely bromelain, papain, and pancreatin, were utilized to produce distinct hydrolysates, which were subsequently evaluated for their antioxidant capacity and fungal growth inhibition, two key indicators of biotechnological interest.

This work supports the broader application of enzyme-assisted extraction and targeted hydrolysis as enabling tools in the sustainable valorization of plant biomasses.

## 2. Results and Discussion

### 2.1. Protein Extraction and Molecular Characterization

Protein extraction from agri-food products is often hindered by the supramolecular organization, which can be significantly altered during industrial processing, especially if heat treatment is required. These structural changes frequently lead to novel molecular interactions that reduce extractability and complicate downstream or scaled-up applications. Consequently, tailored extraction strategies become necessary to address these challenges [7].

Our extraction strategy was designed to prioritize functional recovery and enzymatic digestibility, to allow the production of bioactive peptide fractions for biotechnological applications. As a preliminary step, the grounded tomato seed, which is characterized by a high lipid content [18], was defatted to eliminate potential hydrophobic interferences during protein extraction and to enhance protein accessibility. By performing defatting using an automated extraction system (Dionex ASE, ThermoFisher, Waltham, MA, USA), the lipid fraction was quantitatively removed and measured, yielding a lipid content of 28.22 ± 0.02%, which is in agreement with the literature [18]. This step was essential to prevent emulsification phenomena and steric hindrance caused by oil droplets, which could otherwise compromise protein–solvent interactions and reduce extraction efficiency.

The total protein content of the defatted flour, determined by the Kjeldahl method, was 34.71 ± 0.02%, serving as a reference value for optimizing the different extraction methods and selecting the optimal condition for achieving the highest protein solubility.

**Figure 1:**
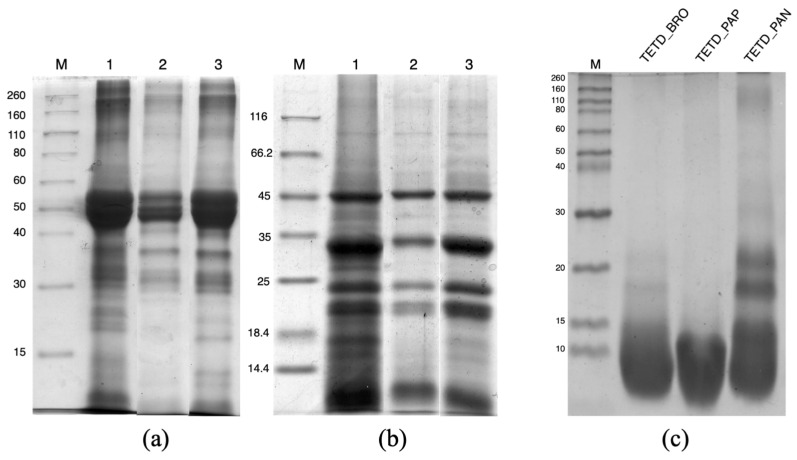
SDS-PAGE of tomato seed protein extracts under different conditions. Panel (**a**) shows the protein profile under non-reducing conditions. Panel (**b**) shows the corresponding samples under reducing conditions. In both panels (12.5% polyacrylamide gel concentration), the lanes are as follows: M—molecular weight marker; 1—SDS-denatured protein extract; 2—mild protein extract; 3—alkaline protein extract. Panel (**c**) displays the electrophoretic profile of enzymatically digested total extract tomato (TETD) under reducing conditions (17% polyacrylamide gel concentration). BRO = Bromelain; PAP = Papain; PAN = Pancreatin.

For a first visualization of the protein profile, the defatted tomato seed flour was directly solubilized in Laemmli buffer [19] containing SDS and a reducing agent (when indicated), boiled for complete protein denaturation, and then loaded onto an SDS-PAGE (Figure 1a,b, lane 1). The electrophoretic pattern obtained from this sample served as a reference, representing the complete set of extractable polypeptides, as it encompassed both quantitative yield and qualitative diversity. However, it was not compatible with further biochemical applications due to the chemistry of the extraction buffer and the presence of surfactants.

To explore biocompatible extraction methods, two main conditions were tested: a mild extraction at pH 7.4 and an alkaline extraction at pH 11.0, performed in the same buffer. From the quantification using the Bradford dye-binding method [20], the mild extraction yielded 48.86 ± 2.75 mg of proteins/g of flour, due to the solubilization of mostly proteins that were not aggregated or loosely associated within the seed matrix. The alkaline extraction allows for greater protein recovery (139.77 ± 10.39 mg of proteins/g of flour), corresponding to over 40% of the total protein content as referred to the Kjeldahl data. The effectiveness of the alkaline extraction is consistent with the literature reports [13,21]. The comparison of the electrophoretic profiles of proteins extracted under mild and alkaline conditions (Figure 1a,b, lanes 2 and 3) confirms that pH 11.0 enables a greater quantitative recovery. Moreover, in both conditions, the protein pattern is similar to the reference SDS-denatured protein extract. In non-reducing conditions (Figure 1a), molecular weight bands ranging between 50 and 40 kDa are more abundant in the alkaline extract. These proteins, stabilized by covalent interactions and separated into subunits with molecular weights of around 30 and 20 kDa under reducing conditions (Figure 1b), confirm their abundance in the pH 11.0 extract. Additionally, it is notable that with mild conditions, there is a lower extraction yield of proteins/polypeptides with molecular weights at approximately 25 and 15 kDa.

To further characterize the biochemical behavior of tomato seed proteins, we investigated the molecular interactions that govern their aggregation and potential association with the surrounding matrix. Based on this rationale, and to probe these interactions, we applied a differential solubility approach by performing additional extractions, adding 6 M urea (a chaotropic agent) and 10 mM DTT (a reducing agent) [22]. These treatments were not intended for functional applications but were crucial for revealing the types of interactions that stabilize the insoluble protein fractions. As expected, the results show an increase in solubility, as referred to the extract obtained in mild conditions, in the presence of urea (114.84 ± 2.87 mg of proteins/g of flour) and with the addition of urea plus DTT (126.05 ± 3.83 mg of proteins/g of flour). The major increased solubility that is observable in the chaotropic condition suggests a major contribution of hydrophobic interactions in the stabilization of the protein aggregates. The further increment of the solubilized protein by the addition of the reducing agent indicates the contribution of the disulfide bridges in the supramolecular aggregation of tomato seed proteins. These findings confirm that seed storage proteins are structurally stabilized by both non-covalent and covalent interactions within cellular storage organelles, requiring specific physicochemical conditions to be effectively disrupted. Furthermore, they suggest an overall molecular rearrangement during the industrial technological processing of the tomato.

Since alkaline extraction allows for greater protein recovery and, after pH correction, is compatible with proteolytic activity, this approach was selected for downstream applications. The extract obtained under these conditions was named Total Extract Tomato (TET).

### 2.2. Enzymatic Hydrolysis and Molecular Characterization of Peptides

The alkaline extract (TET) was subjected to proteolytic digestion using three commercial enzymes: bromelain (BRO), papain (PAP), and pancreatin (PAN), each characterized by distinct catalytic mechanisms, to obtain different peptide mixtures. Bromelain, a cysteine protease, has broad substrate specificity, cleaving after neutral and basic amino acids even within structured domains. Papain, although also a cysteine protease, favors cleavage after hydrophobic residues and appears less effective on tightly folded globular proteins. Pancreatin, a mixture of digestive serine proteases (e.g., trypsin, chymotrypsin), is effective on exposed peptide bonds but may be hindered by plant-specific structural features such as disulfide bonding or glycosylation.

The extent of protein hydrolysis was assessed using SDS-PAGE and OPA assay. The electrophoretic analysis (Figure 1c) revealed frank differences in the degree of proteolysis among the three enzyme treatments. The bromelain hydrolysate (TETD_BRO) showed a near-complete disappearance of the extracted protein, accompanied by a broad, diffuse accumulation at low molecular weights, indicating extensive proteolysis and the formation of small peptides. A similar trend can be observed for the papain hydrolysate (TETD_PAP), where the accumulation of small polypeptide fragments is evident. In contrast, pancreatin hydrolysate (TETD_PAN) retained visible bands with a molecular weight of about 20 kDa, indicating a more limited enzymatic digestion and the persistence of mid-sized protein fragments.

**Figure 2:**
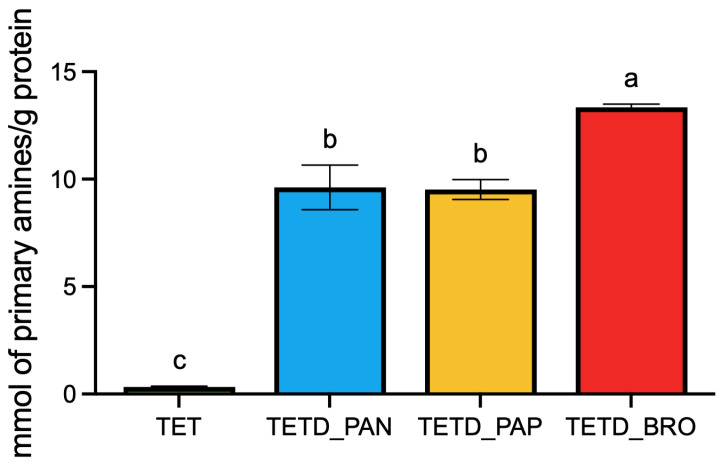
Quantification of free amino groups in undigested and enzymatically digested protein extracts using the o-phthaldialdehyde (OPA) assay. The concentration of free amino groups is reported as mmol of primary amines per gram of extracted proteins, based on a calibration curve realized with L-glutamic acid. The data are expressed as mean ± standard deviation (*n* = 3). Statistical analysis was performed using one-way ANOVA followed by Tukey’s post hoc test. Different letters indicate statistically significant differences among groups (*p* < 0.05).

These results were confirmed by OPA quantification (Figure 2), which measures the release of primary amino groups during hydrolysis. The results show that the TETD_BRO sample presents the highest amount of α-amino nitrogen per g of extracted protein (13.35 ± 0.15), followed by TETD_PAP and TETD_PAN (respectively, 9.61 ± 1.04 and 9.51 ± 0.46; *p*-value = 0.9962).

**Figure 3:**
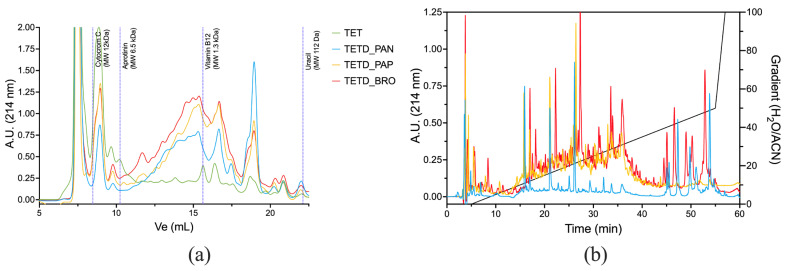
Chromatographic profiles of enzymatically digested protein extracts. Panel (**a**): SEC-HPLC elution profiles monitored at 214 nm with a flow of 0.5 mL/min. The dashed vertical lines indicate elution volumes of molecular weight standards. Panel (**b**): RP-HPLC chromatograms of the same samples, monitored at 214 nm, with the acetonitrile gradient (H_2_O/ACN, % *v*/*v*) shown by the black line. Samples: TET (green), TETD_PAN (blue), TETD_PAP (yellow), and TETD_BRO (red).

The peptide mixtures were analyzed using Size Exclusion (SEC-HPLC) and Reverse Phase (RP-HPLC) chromatography to provide complementary insights into the molecular size and hydrophobicity of the peptides generated by enzymatic digestions. The SEC-HPLC chromatograms (Figure 3a) show an increased presence of peptides in the enzymatic digests compared to the original extract. TETD_PAN showed a significant shift toward low molecular weight, with peptide sizes ranging from ~1.3 kDa down to 112 Da (corresponding to peptides of 11–13 amino acids in length and free amino acids). TETD_BRO and TETD_PAP exhibited similar peak distributions, though BRO showed a higher amount of hydrolysis products. RP-HPLC profiles (Figure 3b) highlight the emerged differences in peptide polarity. TETD_PAN showed a profile characterized by the presence of more polar peptides (eluted between 10–20% H_2_O/Acetonitrile mobile phase) and peaks eluted under hydrophobic conditions (40–50% gradient). TETD_PAP presented an intermediate peak pattern, with peaks eluted between 10 and 40% of H_2_O/Acetonitrile mobile phase. TETD_BRO was characterized by a comparable TETD_PAP peak profile within the 10–40% gradient range. Still, it also exhibited hydrophobic species peaks, indicative of a peptide population enriched in hydrophobic sequences, such as those containing aromatic or aliphatic amino acids, which are also visible in the TETD_PAN sample.

Taken together, the results of the HPLC characterization suggest that the enzymatic activity of bromelain is more effective in generating a more diverse peptide population.

### 2.3. Antioxidant Properties and Polyphenol Content

The antioxidant capacity of the undigested and the digested extracts was evaluated using three complementary assays: ABTS, DPPH, and FRAP (Table 1). Together, these tests provided a multidimensional view of the radical scavenging and reducing capacity of the three generated peptide mixtures. Overall, enzymatic treatments led to a marked improvement in antioxidant activity compared to the non-hydrolyzed protein extract (TET), demonstrating that hydrolysis can effectively unlock antioxidant functionalities from tomato seed proteins. However, there are some differences in the results obtained with the three assays used.

To fully understand the meaning of these results, it is necessary to consider the differing chemical mechanisms of the antioxidant assays [23,24,25].

The ABTS assay evaluates radical scavenging activity via both hydrogen atom transfer and single electron transfer mechanisms and is sensitive to a broad spectrum of hydrophilic and lipophilic antioxidants [26]. Among the hydrolysates, TETD_PAN exhibited the highest ABTS activity. This is consistent with the RP-HPLC profile, where TETD_PAN showed the presence of various peaks eluted at both low and high concentrations of solvent, showing a good radical-quenching potential. Additionally, both digested samples, TETD_BRO and TETD_PAP, showed a higher radical-quenching capacity compared to TET. However, the difference between TETD_BRO and TETD_PAP was not statistically significant (*p*-value = 0.5165). Both hydrolysates are rich in peaks eluted in an intermediate hydrophobic condition. This chemical composition may explain their reduced ability to scavenge the ABTS radical cation.

In contrast, the DPPH assay is based mainly on hydrogen atom donation and is less responsive to hydrophilic antioxidants [27], which may partly explain the divergent ranking of the samples compared to the ABTS assay. In this test, TETD_BRO exhibited the highest activity, consistent with its RP-HPLC profile, which is rich in hydrophobic peptides. TETD_PAP also showed relatively high DPPH activity, in agreement with its moderately hydrophobic peptide profile. In contrast, TETD_PAN showed the lowest DPPH value, reflecting its higher content of polar peptides, which are less reactive under the non-aqueous conditions of this assay.

The FRAP assay, on the other hand, does not rely on radical quenching but instead measures the ability of antioxidants to reduce Fe^3+^ to Fe^2+^, emphasizing electron transfer capacity [28]. All the tested samples showed similar FRAP values, ranging from 122.8 to 138.8 µmol TE/g of flour. The results indicate that, after enzymatic digestion, there is no observable enhancement in reducing capacity.

Although the extraction and hydrolysis processes were not specifically designed to extract phenolic compounds, all samples showed detectable total phenolic content (TPC) values (Table 1). The total phenolic content of the hydrolysates ranged from 2.29 ± 0.10 to 2.74 ± 0.11 mg GAE/g of flour. The highest TPC was observed in TETD_PAN. However, the absolute levels are not very different from the values of the undigested extract. These results suggest that the observed antioxidant properties were primarily attributable to the released peptides, rather than to phenolic co-extractives.

Taken together, the results showed that TETD_BRO consistently stood out for its balanced performance across multiple assays, including strong DPPH activity. Combined with its broad molecular weight and hydrophobic features of the peptides profile, TETD_BRO was selected for further evaluation of antifungal bioactivity, given its potential to interact with biological membranes and affect redox-sensitive processes in phytopathogens.

Given the planned antifungal tests, we wanted to address the presence in TET of compounds other than proteins/peptides that could hamper the results, such as carbohydrates. We detected a substantial amount of co-extracted soluble sugars (12.72 ± 0.17 mg/mL), which posed a risk of introducing bias when testing the hydrolyzed sample in biological assays by serving as a potential carbon source for fungal growth.

### 2.4. Evaluation of Antifungal Activity of the Bromelain Hydrolyzed Sample

To eliminate the potential interfering presence of soluble sugar and ensure that antifungal effects could be confidently attributed to the hydrolysis products, TET was subjected to protein precipitation before enzymatic hydrolysis. The pellets were then resuspended in buffer, adjusted to the optimal pH for each enzyme. This step was performed only for the preparation of samples used in antifungal tests to ensure the specificity and interpretability of antifungal assays.

The antifungal activity was evaluated against two important fungal species: *Fusarium graminearum*, a major pathogen of wheat and maize, and *F. oxysporum* f.sp. *lycopersici*, an important pathogen of tomato. To assess its inhibitory effect, TETD_BRO was applied at varying concentrations to evaluate the dose-dependency of the response and to compare the sensitivity of the two fungal species.

**Figure 4:**
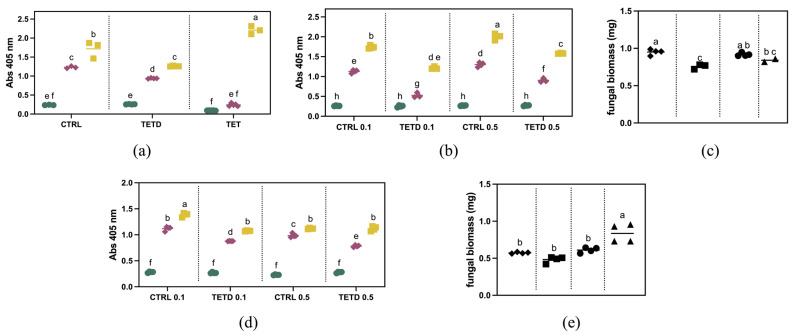
Antifungal activity evaluation of undigested and bromelain-derived hydrolysate at different concentrations against *Fusarium graminearum* (Panels (**a**,**b**)) and *F. oxysporum* f.sp. *lycopersici* (Panel (**d**)). Green = 1 day of growth; Red = 3 days of growth; Yellow = 5 days of growth. Panels (**c**,**e**): Dry weight after 5 days of growth of *Fusarium graminearum* colonies and *F. oxysporum* f.sp. *lycopersici* colonies, respectively. Diamond = Control 0.1 mg/mL, Squares = TETD 0.1 mg/mL, Circles = Control 0.5 mg/mL, Triangles = TETD 0.5 mg/mL. The data are expressed as mean ± standard deviation (*n* = 4). Statistical analysis was performed using one-way ANOVA followed by Tukey’s post hoc test. Different letters indicate statistically significant differences among groups (*p* < 0.05).

To explore the bioactivity of bromelain digested and undigested samples against *F. graminearum*, they were initially tested at a concentration of 2 mg/mL. As shown in Figure 4a, after five days, the fungal growth is lower than the control, corresponding to a ~27% reduction. Similarly, after three days, a ~24% reduction was observed. This trend is not observable in the presence of the undigested sample. These results suggest that in TETD_BRO, peptides with antifungal activity are present. The digested sample was further tested at a lower concentration (Figure 4b). The results showed that at 0.1 mg/mL, the fungal growth was inhibited by approximately 54% after 3 days and 30% after 5 days. At 0.5 mg/mL, the inhibition was about 31% at 3 days and 21% at 5 days, suggesting a dose-dependent sensitivity to TETD_BRO. The results observed via optical density measurements were also confirmed by the evaluation of biomass accumulation (Figure 4c), which showed a ~20% reduction at 0.1 mg/mL and a more limited effect of about 8% at 0.5 mg/mL.

The antifungal activity of bromelain-generated peptides was also tested against *F. oxysporum* f.sp. *lycopersici*. As shown in Figure 4d, a concentration of 0.1 mg/mL reduced fungal growth by about 22% at both 3 and 5 days. At 0.5 mg/mL, the inhibition was approximately 21% after 3 days, but no difference was detectable at 5 days compared to the control (*p*-value = 0.06065). The biomass measurements at 5 days (Figure 4e) show a slight decrease for the 0.1 mg/mL treatment compared to the control, although it is not statistically different (*p* = 0.2775). Regarding the 0.5 mg/mL treatment, no reduction in biomass was observed.

Taken together, these findings suggest a species-specific susceptibility, possibly linked to differences in membrane composition or defense mechanisms. The differential sensitivity may be attributed to the host-specific pathogenicity of *F. oxysporum* f. sp. *lycopersici*, which is highly adapted to *Solanum lycopersicum* L., potentially endowing it with enhanced resistance mechanisms against exogenous antimicrobial peptides or proteolytic enzymes [29,30].

## 3. Materials and Methods

### 3.1. Materials

Tomato pomace was sampled at a full-scale tomato cannery (Ravarino, Modena, Italy) during the production season 2023. Then the seeds were separated from the peels and successively dried at 50 °C.

Fungal strains were obtained from USDA-NRRL (Kindly provided by Dr. Kerry O’Donnell).

All reagents, unless stated otherwise, were obtained from Merck (Darmstadt, Germany).

### 3.2. Defatting

Tomato seed samples were defatted using a Dionex ASE 350 Accelerated Solvent Extractor (ThermoFisher, Waltham, MA, USA), following a modified protocol based on Application Note 329 from the “Accelerated Solvent Extraction Applications Summary” (ThermoFisher, Waltham, MA, USA). Pentane was used as the solvent for extraction. The tomato seeds were milled using an A 11 basic Analytical Mill (IKA, Staufen, Germany) and directly utilized for defatting. Exactly 30 g of the milled seeds were placed into 66 mL stainless steel extraction cells. The extraction was performed under the conditions reported in Table 2.

After extraction, the pentane containing dissolved lipids was collected and subjected to solvent removal using a rotary evaporator (Rotavapor, Büchi, Flawil, Switzerland). The weight of the lipid residue was measured using an analytical balance. Gravimetric quantification was performed by comparing the weight of the empty collection flask before and after solvent evaporation.

### 3.3. Protein Extraction and Quantification

#### 3.3.1. Protein Extraction

The total protein content was determined on defatted samples using the Kjeldahl method, according to AOAC Official Method 978.04 (AOAC, 2005) [31], using a nitrogen-to-protein conversion factor of 6.5.

The extraction of the protein fraction was performed in 50 mM sodium phosphate buffer at pH 7.4 for the mild extraction and in the same buffer at pH 11 for the alkaline extraction [21]. Grounded and defatted seeds were suspended in the appropriate buffer at a 1:30 (*w*/*v*) ratio and stirred for 1 h at 4 °C. The suspension was then centrifuged at 11,000× *g* for 20 min at 4 °C to collect the clear supernatant.

Before the evaluation of the antifungal activity, Total Extract of Tomato at pH 11 (TET) was subjected to precipitation with ammonium sulfate to concentrate the proteins and remove coextracted compounds (in particular the carbohydrate fraction). Precipitation was carried out at 4 °C by adding ammonium sulfate to a final concentration of 90% (*w*/*v*) while stirring the sample for 30 min. The precipitated proteins were collected by centrifugation at 11,000× *g* for 20 min at 4 °C. The resulting pellet was resuspended in the original extraction buffer (50 mM sodium phosphate buffer at pH 11) in 2/3 of the original extraction volume before the bromelain digestion.

The TET protein quantification was carried out by a dye-binding method, the Bradford [20].

#### 3.3.2. Differential Solubility

To investigate the structural organization and conformational stability of tomato seed proteins, we applied a differential solubility approach [22,32]. Specifically, 150 mg of tomato seed flour was suspended in 4.5 mL of 50 mM sodium phosphate buffer at pH 7.4, in the presence of 6 M urea and 6 M urea plus 10 mM dithiothreitol (DTT), when indicated, maintaining a 1:30 (*w*/*v*) sample-to-buffer ratio. The protein concentration in the supernatant was quantified using the Bradford dye-binding assay [20]. The results are expressed as mean ± standard deviation of three independent extractions.

### 3.4. Protein Digestion

Total Extract of Tomato at pH 11 (TET) was aliquoted and subjected to enzymatic digestion to obtain hydrolyzed extracts (TETD) using three commercial enzymes (Merck, Darmstadt, Germany): bromelain (B4882), papain (P4762), and pancreatin (P1625). After adjusting the pH of the protein extract to match the optimal pH of the enzymes (papain and bromelain pH 7.0; pancreatin pH 8.0), digestion was performed by directly suspending the enzyme in the extract, using a protein/enzyme ratio of 1:50 (*w*/*w*), and agitating the suspension for 24 h at 37 °C. At the end of the digestion, the enzymes were thermally inactivated (70 °C for 30 min), following centrifugation (5000× *g*, 20 min at 25 °C). The supernatants were quantified by a dye-binding method [20].

### 3.5. Peptide Quantification

The amount of peptide generated by enzymatic hydrolysis was evaluated by performing the α-amino nitrogen quantification by the O-Phthaldialdehyde (OPA) method. Primary amines were quantified by the OPA assay [33] with slight modifications. The OPA reagent was prepared by mixing 0.5 mL of OPA reagent (40 g/L in ethanol), 2.5 mL of SDS (10% *w*/*v*), 12.5 mL of borate buffer (0.1 mol/L), 0.5 mL of 2-mercapto-ethansulfonic acid solution (200 g/L), 1.25 mL of Triton X-100 (100 g/L), 7.75 mL of water. Samples (8 µL) and standards were added to a 96-well plate, followed by 232 µL of OPA working solution. After incubation at 30 °C for 10 min in the dark, absorbance was measured at 335 nm by using a microplate reader Tecan Infinite M Nano^+^ (Tecan Trading AG, Männedorf, Switzerland). The results were expressed as mmol of primary amine per gram of extracted protein, determined through a calibration curve realized with L-glutamic acid prepared in 0.5 M perchloric acid.

### 3.6. Protein and Peptide Characterization

#### 3.6.1. Electrophoretic Characterizations

For total protein analysis, 1 mg of milled tomato seed powder was resuspended in 50 μL of 0.5 M Tris-HCl buffer (pH 6.8) containing 0.4% SDS and mixed with 50 μL of de-naturing Laemmli buffer (0.125 M Tris-HCl, pH 6.8, 50% glycerol, 1.7% SDS, and 0.01% bromo-phenol blue (*w*/*v*)), with 1% (*v*/*v*) 2-mercaptoethanol (2-ME) added when specified. The suspension was heated at 100 °C for 10 min. The separation was performed on a 12.5% polyacrylamide gel [19].

For the electrophoretic analysis of undigested and hydrolyzed protein extracts, 50 μL of the sample was mixed with 50 μL of the same denaturing buffer described above and heated at 100 °C for 10 min. The sample was then centrifuged, and the supernatant was loaded onto a gel, 12.5% polyacrylamide concentration for undigested extract and 17% polyacrylamide for hydrolyzed extracts. In the latter case, at the end of the runs, gels were fixed in 10% trichloroacetic acid (TCA) for 1 h under shaking at room temperature.

All SDS-PAGE analyses were conducted at pH 8.8 using a running buffer composed of 0.025 M Tris-HCl, 0.192 M glycine, and 0.1% (*w*/*v*) SDS, in a Miniprotean II cell system (Bio-Rad Laboratories, Hercules, CA, USA) and stained with Coomassie Blue. Low-range SDS-PAGE standards (Bio-Rad, Hercules, CA, USA) were used for determining the relative molecular weight.

#### 3.6.2. Chromatographic Characterizations

**Size exclusion high-performance liquid chromatography (SE-HPLC)** was performed on the digested samples in a Superdex Peptide 10/300 GL column (Cytiva Europe GmbH, Milano, Italy) fitted on a chromatographic apparatus composed of a Waters 600E multi-solvent delivery system and a Waters 2487 Dual λ Absorbance Detector (Waters, Sesto San Giovanni, Italy) collecting chromatograms at 214 nm. The mobile phase was 50 mM sodium phosphate buffer pH 8.0, with a flow of 0.5 mL/min. All samples were initially centrifuged at 13,000× *g* and then filtered through a 0.22 μm syringe filter.

**Reverse phase exclusion high-performance liquid chromatography (RP-HPLC)** were performed in a SIMMETRY300 C18 (5 μm) (4.6 × 250 mm) column (Waters) fitted on a LC-4000 Series HPLC system (Jasco Corporation, Tokyo, Japan), equipped with AS-4050 General Purpose HPLC Autosamplers and using an MD-4010 PDA detector. Separations were run at 0.8 mL/min, mixing solution A (water with 0.1% TFA (*v*/*v*)) and solution B (acetonitrile with 0.1% TFA (*v*/*v*)) as reported in Table 3.

### 3.7. Antioxidant Capacity

#### 3.7.1. ABTS Scavenging Assay

The trapping capacity of cationic-free radicals was evaluated using the method of radical 2,2′-azino-bis(3-ethylbenzothiazoline-6-sulfonic acid) diammonium salt (ABTS) bleaching described by Re et al. [26] following the procedure described by Martinez-Saez et al. [34] for its use in a microplate. ABTS stock solution was prepared by mixing the ABTS radical and potassium persulfate and incubated for 16 h in the dark at room temperature. Subsequently, the ABTS working solution was prepared by diluting a stock solution of 10 mM sodium phosphate buffer at pH 7.4 to 1:75 (*v*/*v*) and adjusting the final absorbance to 0.7 ± 0.02 at 734 nm. The assay was performed by mixing 20 μL of the samples with 180 μL of ABTS working solution in a 96-well microplate. Absorbance changes at 734 nm were recorded for 30 min at 25 °C in Tecan Infinite M Nano^+^ microplate reader, with readings every 5 min. The results were expressed as mmol of Trolox equivalents (TE) per g of flour, determined using a calibration curve [35]. All measurements were performed in triplicate.

#### 3.7.2. Determination of Radical Scavenging Capacity Against DPPH

The total antioxidant capacity was evaluated by performing the DPPH assay. In the presence of an antioxidant, the DPPH radical can accept an electron or hydrogen radical to become a stable molecule without dimerization [36]. When this occurs, the violet color of DPPH^•^ decays, and the absorbance change can be followed spectrophotometrically at 517 nm [37]. In short, a solution of 100 μM DPPH in methanol was prepared, with adjustments made to attain an absorbance reading of 0.7 ± 0.02 at 517 nm. Each well of a 96-well microplate contained 192 µL of DPPH and 8 µL of sample. After incubation for 60 min in the dark, the absorbance of the reaction mixture was measured at 517 nm using Tecan Infinite M Nano^+^ microplate reader. The results were expressed as mmol of Trolox equivalents (TE) per g of flour, determined through a calibration curve. All measurements were performed in triplicate.

#### 3.7.3. Determination of the Ferric Reducing Antioxidant Power (FRAP)

The total antioxidant capacity was also assessed by performing the FRAP (Ferric Reducing Antioxidant Power) assay, which is based on the ability of antioxidants to reduce the ferric–TPTZ (Fe^3+^–2,4,6-tripyridyl-s-triazine) complex to its ferrous form (Fe^2+^–TPTZ) under acidic conditions [28]. The resulting Fe^2+^–TPTZ complex develops an intense blue color that was monitored spectrophotometrically at 593 nm [23].

The FRAP reagent was freshly prepared by mixing 25 mL of 0.3 M acetate buffer (pH 3.6), 2.5 mL of 10 mM TPTZ dissolved in 40 mM HCl, and 2.5 mL of 20 mM FeCl_3_·6H_2_O. The final solution was kept at 37 °C before use. Each well of a 96-well microplate contained 290 µL of FRAP reagent and 10 µL of sample or standard. The absorbance was measured at 593 nm every 20 s for 10 min using a Tecan Infinite M Nano^+^ microplate reader thermostat at 37 °C. A reagent blank and a sample blank were included in each run. Sample dilutions were adjusted to yield final absorbance values within the linear range of the standard curve. The results were expressed as mmol of Trolox equivalents (TE) per gram of flour, calculated from a standard curve (25–800 µM). All measurements were performed in triplicate.

### 3.8. Total Phenolic Compound

To measure the amount of Total Phenolic Compounds (TPC) in the extract and digested samples the Folin–Ciocalteu reagent [38] was used, adapting the assay from Contini et al. [39]. Ten microliters of the samples were incubated at room temperature after being suspended in 150 μL of Folin–Ciocalteu (Sigma, Setagaya City, Tokyo, Japan) reagent for three minutes. 50 μL of 0.28 M sodium carbonate was then added, and the mixture was incubated for an additional two hours at 37 °C. The absorbance was measured at 735 nm after the incubation in a Tecan Infinite^®^ M Nano^+^ microplate reader. For each sample, a sample blank and a reagent blank were also examined. The results were expressed as milligrams of gallic acid equivalent (GAE) per gram of sample, determined using a calibration curve. All measurements were performed in triplicate.

### 3.9. Sugar Quantification

The quantification of total sugar in samples was estimated using an anthrone-based method [40], as described by Leyva et al. [41], with slight modifications. The assay was performed by adding 200 µL of samples and standards to a 1 mL of anthrone reagent solution (2 g in 1 L of H_2_SO_4_), absorbance level was analyzed using Lamba2 Perkin Elmer (PerkinElmer, Waltham, MA, USA) after 17 min of incubation at 90 °C. Final concentration of sugar in samples was calculated through standard curve from 0.01 mg/mL to 0.2 mg/mL of D-glucose.

### 3.10. Antifungal Fungal Assay

Conidia of *Fusarium graminearum* and *F. oxysporum* f.sp. *lycopersici* [16] were used to test the growth inhibition of the extracts following the protocol described in De Benedetti et al. [16]. One thousand conidia were added to 2/3 PDB medium and 1/3 medium containing either digested or undigested protein extracts, or the control.

Briefly, all experiments were performed with at least 4 biological replicates and repeated twice in 96-well plates, using the different extract concentrations: 0.1, 0.5 and 2 mg/mL.

Fungal conidia were let to germinate at 25 °C for a period of 5 days. Absorbance measurement was used as a proxy for growth and compared to the dried weight of the generated mycelium in the wells. Reads were performed during the growth period in the plates.

Absorbance was measured at 405 nm with Tecan Infinite^®^ M Nano^+^ microplate reader. To assess the dry weight of fungal colonies, plates were loaded into a freeze-drier (ALPHA 2-4 LO freeze dryer, Martin Christ GmbH, Osterode am Harz, Germany), and the dried mycelium was weighed individually using a precision scale.

### 3.11. Statistical Analysis

The data are presented as mean ± standard deviation. Statistical differences among groups were evaluated using ANOVA. When a factor resulted significantly different (*p*-value < 0.05), Tukey’s HSD post hoc test was applied using GraphPad Prism version 10.5.0 (GraphPad Software, San Diego, CA, USA).

## 4. Conclusions

The explicit objective of this study was to establish experimental pipelines enabling the conversion of tomato industry by-products into bioactives that are relevant for plant protection.

By optimizing the protein extraction method from the by-product, we were able to understand how the proteins clump together. This aggregation state is crucial because it influences the accuracy and quantity of the downstream final results. Two critical elements stand out: the hydrophobic interactions, which largely stabilize protein aggregates, and the disulfide bridges, which play a part in the extensive clumping of tomato seed proteins, hampering the protein solubility and recovery. Although mild extraction conditions (pH 7.4) allow recovery of the most soluble protein fraction, alkaline extraction (pH 11.0) emerges as the most effective and functionally relevant condition for achieving the highest protein extraction yield, enabling the use of TET for subsequent efficient enzymatic hydrolysis. The use of bromelain, a relatively inexpensive industrial enzyme, resulted in a greater quantity of peptides, a broader spectrum of molecular weights and polarity/hydrophobicity profiles, high DPPH radical scavenging activity, and moderate ABTS radical scavenging activity. Consequently, it was chosen for further antifungal evaluations. In summary, the bromelain-derived hydrolysate demonstrated clear and concentration-dependent antifungal activity against both *F. graminearum* and *F. oxysporum* f.sp. *lycopersici*, with a stronger effect observed in the former. Our results confirm the potential efficacy of enzymatic plant-derived antifungal peptides in sustainable plant protection strategies. Despite their diverse modes of action, including membrane disruption and interference with cellular mechanisms, they represent a class of compounds effective in controlling fungal diseases.

It is worth noting that the level of bioactivities observed under the adopted experimental conditions was not comparable in intensity to the effects achieved by specific conventional treatments. As a matter of fact, since the conidia treatments were conducted using a mixture of peptides from enzymatic digestion, we cannot exclude the possibility of a dilution effect, where inactive peptides might have reduced the apparent efficacy of active ones. Further studies are needed to fractionate the peptide mixture and identify specific effective peptides with defined antifungal activity and investigate their mechanisms of action. This approach represents a key strategy to developing a viable alternative to conventional chemical treatments.

## Figures and Tables

**Table 1:** Antioxidant activity and total phenolic content of tomato seed non-hydrolyzed and hydrolyzed protein extract. The results are expressed as means ± standard deviations (*n* = 3). ABTS, DPPH, and FRAP values are expressed as µmol Trolox equivalents (TE) per gram of flour; TPC is expressed as mg gallic acid equivalents (GAE) per gram of flour. Different letters indicate statistically significant differences (*p* < 0.05).

Sample	ABTS μmol TE/g	DPPH μmol TE/g	FRAP μmol TE/g	TPC mg GAE/g
TET	38.33 ± 0.66 c	4.12 ± 0.82 c	138.8 ± 4.68 a	2.34 ± 0.25 b
TETD_PAN	56.73 ± 1.56 a	5.92 ± 1.48 bc	122.8 ± 1.73 b	2.74 ± 0.11 a
TETD_PAP	43.43 ± 2.59 b	8.61 ± 1.77 b	135.8 ± 2.64 a	2.67 ± 0.15 ab
TETD_BRO	41.79 ± 1.11 b	12.2 ± 1.52 a	135.5 ± 2.95 a	2.29 ± 0.10 b

**Table 2:** Extraction condition used for the defatting by using a Dionex ASE 350 Accelerated Solvent Extractor (ThermoFisher).

Parameter	Condition
Temperature	50 °C
Heat Time	6 min
Static Time	5 min
Flush Volume	100%
Purge Time	60 s
Static Cycles	3
Total Extraction Time (per sample)	24 min

**Table 3:** RP-HPLC gradient.

Time (min)	Solvent A (%)	Solvent B (%)	Condition
5	100	-	Isocratic
55	50	50	Linear gradient
58	-	100	Linear gradient
62	-	100	Isocratic
64	100	-	Linear gradient
80	100	-	Isocratic

## Data Availability

The data presented in this study are available on request from the corresponding authors.

## Abbreviations

The following abbreviations are used in this manuscript:

SDS-PAGE	Sodium Dodecyl Sulfate Polyacrylamide Gel Electrophoresis
SEC-HPLC	Size Exclusion High Performance Liquid Chromatography
RP-HPLC	Reverse Phase High Performance Liquid Chromatography
DPPH	2,2-Diphenyl-1-picrylhydrazyl
ABTS	2,2′-Azino-bis(3-ethylbenzothiazoline-6-sulfonic acid
FRAP	Ferric Reducing Antioxidant Power
TPC	Total Phenolic Content
TET	Total Extract of Tomato
TETD	Total Extract of Tomato Digested
BRO	Bromelain
PAP	Papain
PAN	Pancreatin
OPA	o-Phthaldialdehyde
GAE	Gallic Acid Equivalents
TE	Trolox Equivalents
PDB	Potato Dextrose Broth
